# Supplementary material for: Anthropometric and neurocognitive consequences of Campylobacter, enterotoxigenic Escherichia coli, and norovirus: A systematic review
Source: PLoS Negl Trop Dis. 2025 Nov 10;19(11):e0013293. doi: 10.1371/journal.pntd.0013293 (PMC12622849; doi:10.1371/journal.pntd.0013293)
Supplement: S1 Table — (DOCX) [file pntd.0013293.s001.docx]

S1 Table. Search terms

|  |  |
| --- | --- |
| **Sequelae terms** | ("Learning Disabilities"[Mesh] OR "Cognition Disorders"[Mesh:NoExp] OR "Cognitive Dysfunction"[Mesh] OR "Learning Disabilit*"[tiab] OR “Cognitive Development”[tiab] OR “Cogniti*”[tiab] OR "Child Development"[Mesh] OR "Growth Disorders"[Mesh] OR "Body Size"[Mesh] OR "child development" OR "postnatal development" OR "post-natal development" OR growth[tiab] OR "Crown Rump Length" OR height OR stunting OR stunted) |
| **Pathogens and disease presentation** | AND ("Campylobacter"[Mesh] OR "Campylobacter Infections"[Mesh] OR Campylobacter*[tiab] OR "Norovirus"[Mesh] OR Norovirus*[tiab] OR "Enterotoxigenic Escherichia coli"[Mesh] OR ETEC[tiab] OR "Enterotoxigenic E*"[tiab] OR "Diarrhea"[Mesh] OR "Dysentery"[Mesh:NoExp]) |
| **Age group terms:** | AND ("Child, Preschool"[Mesh] OR "Infant"[Mesh] OR "Child*"[tw] OR "Infant*"[All Fields] OR "Newborn"[All Fields] OR "Baby"[All Fields] OR "Babies"[All Fields] OR "Neonat*"[All Fields] OR "Pediatric"[tw] OR "Paediatric"[tw]) |
| **Publication date** | AND “1980/01/01”[Date - Publication] : “2024/21/08 [Date - Publication] |
| **Publication type** | NOT (“Editorial”[Publication Type] OR “Letter”[Publication Type] OR “Review”[Publication Type] OR “Case Reports”[Publication Type]) |
