# Supplementary material for: Anthropometric and neurocognitive consequences of Campylobacter, enterotoxigenic Escherichia coli, and norovirus: A systematic review
Source: PLoS Negl Trop Dis. 2025 Nov 10;19(11):e0013293. doi: 10.1371/journal.pntd.0013293 (PMC12622849; doi:10.1371/journal.pntd.0013293)
Supplement: S2 Table — (DOCX) [file pntd.0013293.s002.docx]

S2 Table. Quality reporting scale utilized for studies included in systematic review*

| **Method head** | **Description** | **Score** |
| --- | --- | --- |
| **STROBE Guidelines** | |  |
| **Study design** | Present key elements of study design early in the paper | 1 |
| **Setting** | Describe the setting, locations, and relevant dates, including periods of recruitment, exposure, follow-up, and data collection | 1 |
| **Participants** | (*a*) *Cohort study*—Give the eligibility criteria, and the sources and methods of selection of participants. Describe methods of follow-up  *Case-control study*—Give the eligibility criteria, and the sources and methods of case ascertainment and control selection. Give the rationale for the choice of cases and controls  *Cross-sectional study*—Give the eligibility criteria, and the sources and methods of selection of participants | 1 |
|  | (*b*) *Cohort study*—For matched studies, give matching criteria and number of exposed and unexposed  *Case-control study*—For matched studies, give matching criteria and the number of controls per case |  |
| **Variables** | Clearly define all outcomes, exposures, predictors, potential confounders, and effect modifiers. Give diagnostic criteria, if applicable | 1 |
| **Data sources/** **measurement** | For each variable of interest, give sources of data and details of methods of assessment (measurement). Describe comparability of assessment methods if there is more than one group | 1 |
| **Bias** | Describe any efforts to address potential sources of bias | 1 |
| **Study size** | Explain how the study size was arrived at | 1 |
| **Quantitative** **variables** | Explain how quantitative variables were handled in the analyses. If applicable, describe which groupings were chosen and why | 1 |
| **Statistical** **methods** | (*a*) Describe all statistical methods, including those used to control for confounding | 1 |
|  | (*b*) Describe any methods used to examine subgroups and interactions |  |
|  | (*c*) Explain how missing data were addressed |  |
|  | (*d*) *Cohort study*—If applicable, explain how loss to follow-up was addressed  *Case-control study*—If applicable, explain how matching of cases and controls was addressed  *Cross-sectional study*—If applicable, describe analytical methods taking account of sampling strategy | 1 |
|  | (*e*) Describe any sensitivity analyses |  |
| **Adapted from: von Elm E, Altman DG, Egger M, Pocock SJ, Gøtzsche PC, Vandenbroucke JP. The Strengthening the Reporting of Observational Studies in Epidemiology (STROBE) statement: guidelines for reporting observational studies. J Clin Epidemiol 2008;* ***61****(4): 344-9.* | | |
