# Supplementary material for: Anthropometric and neurocognitive consequences of Campylobacter, enterotoxigenic Escherichia coli, and norovirus: A systematic review
Source: PLoS Negl Trop Dis. 2025 Nov 10;19(11):e0013293. doi: 10.1371/journal.pntd.0013293 (PMC12622849; doi:10.1371/journal.pntd.0013293)
Supplement: S3 Table — (DOCX) [file pntd.0013293.s003.docx]

S3 Table. Quality ratings of included studies

| **Study** | **Country** | **Recruitment Years** | **Study name (if applicable)** | **Adapted STROBE score** |
| --- | --- | --- | --- | --- |
| Amour 2016 | Bangladesh, India, Nepal, South Africa, Tanzania, Brazil, Peru | 2009-2012 | MAL-ED | 8 |
| Black 1984 | Bangladesh | 1978-1979 | Not named | 8 |
| Bray 2019 | Bangladesh | 2007-2010 | GEMS | 5 |
| Caulfield 2017 | Bangladesh, India, Nepal, South Africa, Tanzania, Brazil, Peru | 2009-2014 | MAL-ED | 9 |
| Das 2021 | Bangladesh | 2007-2011 | GEMS | 7 |
| Das 2022 | Bangladesh, India, Pakistan, The Gambia, Mali, Mozambique,  Kenya | 2007-2011 | GEMS | 8 |
| Das 2024 | Bangladesh, India, Pakistan, The Gambia, Mali, Mozambique,  Kenya | 2007-2011 | GEMS | 8 |
| Diaz 2023 | Haiti | 2020-2021 |  | 8 |
| Donowitz 2021 | Bangladesh | 2014-2016 |  | 6 |
| George 2017 | Bangladesh | 2014 |  | 6 |
| George 2023 | Democratic Republic of the Congo | 2018-2019 | REDUCE | 7 |
| González-Fernández 2023 | Pakistan | 2010-2012 | MAL-ED | 8 |
| Haque 2019 | Bangladesh, India, Nepal, South Africa, Tanzania, | 2009-2012 | MAL-ED | 8 |
| Haque 2023 | Bangladesh, Brazil, India, Nepal, Peru, Pakistan, South Africa, Tanzania | 2009-2012 | MAL-ED | 9 |
| Hossain 2023 | Bangladesh, India, Pakistan | 2007-2011 | GEMS | 8 |
| Iqbal 2019 | Pakistan | 2012-2015 |  | 6 |
| Kabir 2022 | Pakistan | 2016-2018 | SEEM | 6 |
| Lee 2013 | Peru | 2002-2006 | Not named but same cohort as Lee 2014 | 8 |
| Lee 2014 | Peru | 2002-2006 | Not named but same cohort as Lee 2013 | 8 |
| Luoma 2023 | Malawi | 2009-2011 | iLiNS‐DYAD‐M | 9 |
| Nasrin 2021 | The Gambia, Mali, Mozambique, Kenya, Pakistan, Bangladesh, India | 2007-2011 | GEMS | 10 |
| Pajuelo 2024 | Peru | 2016-2019 |  | 7 |
| Palit 2022 | Bangladesh, India, Nepal, South Africa, Tanzania, Brazil, Peru |  | MAL-ED | 8 |
| Platts-Mills 2014 | Tanzania | 2009-2012 | MAL-ED | 7 |
| Platts-Mills 2017 | Bangladesh | 2009-2012 | PROVIDE | 7 |
| Rogawski 2018 | Bangladesh, India, Nepal, South Africa, Tanzania, Brazil, Peru | 2009-2012 | MAL-ED | 9 |
| Rouhani 2020 | Peru | 2009-2012 | MAL-ED | 8 |
| Sanchez 2020 | Bangladesh | 2010-2012 | MAL-ED | 8 |
| Scharf 2023 | Brazil, Tanzania, South Africa | 2009-2012 | MAL-ED | 8 |
| Schnee 2018 | Bangladesh | 2011-2014 | PROVIDE | 8 |
