# Supplementary material for: Anthropometric and neurocognitive consequences of Campylobacter, enterotoxigenic Escherichia coli, and norovirus: A systematic review
Source: PLoS Negl Trop Dis. 2025 Nov 10;19(11):e0013293. doi: 10.1371/journal.pntd.0013293 (PMC12622849; doi:10.1371/journal.pntd.0013293)
Supplement: Attachment — Submitted filename: PNTD-D-25-01043_Reviewers comments_JH.docx [file pntd.0013293.s005.docx]

**PNTD-D-25-01043**

This systematic review paper described the anthropometric and neurocognitive of bacterial (Campylobacter and enterotoxigenic E. Col) and norovirus associated diarrhea or asymptomatic infection.

*Campylobacter,* enterotoxigenic *E. Col and* Norovirus are the major diarrhoeal pathogen mostly affected in Asia and Africa and has the impact on diarrhea morbidity and mortality. This systematic review paper has carefully reviewed, analysed and summarized the published research findings on *Campylobacter*, ETEC, norovirus, and their anthropometric and neurocognitive outcome very thoroughly. The introduction, methods, results and the discussion sections are well written This paper has highly public health importance. This review paper deserves for publication.

**The following comments are to review and address/update in the manuscript:**

1. **Title and review paper:** From the title, it is not clear whether the consequences are due pathogenic infection or diarrhea associated with specific pathogens. The authors could consider the title of this review paper as “Anthropometric and neurocognitive consequences of *Campylobacter*, enterotoxigenic *Escherichia coli*, and norovirus associated diarrhea or asymptomatic infection: A systematic review”. This will make the title clear for the reader.
2. **Conclusion, abstract**: The modest outcome (liner growth detriment) is not included in conclusion. The authors can consider to include.
3. **Page 3, L 21-23:** Give reference for the sentence “Shigella------ antibiotic use”
4. **Page 3, L 23-24:** Give reference for the sentence “Norovirus ------ growth faltering”
5. **References:** The references are 39 in the review paper. However, the references numbers are up to 45 in table 1 and table 2. Please include the missing references.
6. **Page 6, Line 91:** Total numbers (n=22) of reported publications/reports and by outcome are confusing in Table 2a. Please check and confirm (as for example, number of reported outcomes for weight gain and wasting are not consistent)
7. **Page 6, L-96:** The reference number 19 is not matching with the text. The reference number will be 15.
8. **Table 2a, wasting outcome:** Please share the page number in table too. Figure S7 has not been shared for review. Only figure 1 is shared.
9. **Figures (as for example, Fig 4A and Fig4B):**  The figures have not been shared for review.
10. **Table 2b.** Please recheck the number reported publications/ outcomes. It is better to add columns and to add sl. No.. for reported publications and outcomes. This will be more convenient and update in the manuscript in there are inconsistent.
11. **Page 7, L 123-125:** The reference number **19** is not matching with the text
12. **Page 7, L 125-128:** Please recheck the references and matched with Table b.
13. **Table 2c:** reference # 23 is not included in table 2c
14. **Co-infection:** The co-infection with other major pathogens could affect the outcome. How, did the authors deal with the co-infection?
